# Size-dependent density of zirconia nanoparticles

**DOI:** 10.3762/bjnano.6.4

**Published:** 2015-01-05

**Authors:** Agnieszka Opalinska, Iwona Malka, Wojciech Dzwolak, Tadeusz Chudoba, Adam Presz, Witold Lojkowski

**Affiliations:** 1 Institute of High Pressure Physics, Polish Academy of Science, Sokołowska 29/37, 01-142 Warsaw, Poland; 2 Faculty of Management, Białystok University of Technology, Wiejska 45A, 15-351 Białystok, Poland

**Keywords:** density, hydrothermal synthesis, hydroxy groups, nanometrology, nanopowders, zirconia

## Abstract

The correlation between density and specific surface area of ZrO_2_ nanoparticles (NPs) was studied. The NPs were produced using a hydrothermal process involving microwave heating. The material was annealed at 1100 °C which resulted in an increase in the average grain size of the ZrO_2_ NPs from 11 to 78 nm and a decrease in the specific surface area from 97 to 15 m^2^/g. At the same time, the density increased from 5.22 g/m^3^ to 5.87 g/m^3^. This effect was interpreted to be the result of the presence of a hydroxide monolayer on the NP surface. A smaller ZrO_2_ grain size was correlated with a larger contribution of the low density surface layer to the average density. To prove the existence of such a layer, the material was synthesized using 50% heavy water. Fourier transform infrared spectroscopy (FTIR) permitted the identification of the –OD groups created during synthesis. It was found that the –OD groups persisted on the ZrO_2_ surface even after annealing at 1100 °C. This hydroxide layer is responsible for the decrease in the average density of the NPs as their size decreases. This study of the correlation between particle size and density may be used to assess the quality of the NPs. In most cases, the technological aim is to avoid an amorphous layer and to obtain fully crystalline nanoparticles with the highest density possible. However, due to the effect of the surface layers, there is a maximum density which can be achieved for a given average NP diameter. The effect of the surface layer on the NP density becomes particularly evident for NPs smaller than 50 nm, and thus, the density of nanoparticles is size dependent.

## Introduction

Zirconium oxide (ZrO_2_) has a wealth of potential applications in the fields of catalysis [1–2], restorative dentistry, high temperature ceramics [3–4], polymer nanocomposites [5–6] and sensors [7]. The characteristics of nanoscale ZrO_2_ (including the mechanical, electrical, chemical, as well as catalytic properties) differ from those of conventional micrometer-sized ZrO_2_. These differences are the result of the unusual properties which occur at the nanoscale and the surface phenomena [8–10]. Using nano-ZrO_2_ as a waveguide host matrix material for light and optical amplification is promising due to its very good chemical and photochemical stability [11–13], high refractive index, and good transparency from the visible to the NIR spectral range [14–16]. Nano-ZrO_2_ is an established, excellent host material for rare earth ions; for example, ZrO_2_:Eu^3+^ is used as a red luminophore [17–18]. When nano-ZrO_2_ is used as a luminescent material, the luminescence intensity increases with crystallite size [17].

The size and surface properties of NPs are also important for toxicology and health applications. The size of the NPs can influence their distribution in the human body and the mechanism of their penetration into the cells and tissues [19]. The chemical composition of the nanomaterial surface has a strong influence on its chemical interaction with tissue [20].

Microcrystalline ZrO_2_ has three polymorphs: monoclinic (m), tetragonal (t), and cubic (c) phases. The monoclinic phase is thermodynamically stable up to 1100 °C and transforms to t-ZrO_2_, which exists in the temperature range 1100–2370 °C, while the cubic phase is found above 2370 °C [21]. However, for nanocrystalline powders, the tetragonal phase can be obtained directly during hydrothermal synthesis.

It was previously observed that density, specific surface area, and phase composition of nano-ZrO_2_ depends on the hydrothermal synthesis conditions. An increase of the synthesis temperature above 260 °C leads to an increase in the nano-ZrO_2_ density. It should be noted that in this case the density was always less than that of bulk ZrO_2_ [22]. The improvement in the density was attributed to the higher crystallinity of the nanoparticles, that is, a smaller amount of amorphous hydroxides were present as the synthesis temperature was increased.

The presence of hydroxy groups on the nanoparticle surface may strongly influence many of their properties such as their chemical reactivity and hydrophilic properties. The hydroxy groups on the NP surface can also operate as effective adsorption sites for organic substances from the atmosphere [23]. Grave and colleagues attributed the increase of the nanopowder luminescence intensity with gain growth to the elimination of surface hydroxy (–OH) groups by heat treatment [24].

The influence of –OH groups on various properties of nano-oxides has been extensively examined [25–28]. Takeda reported that –OH groups on a SiO_2_ surface can function as effective reactive sites [23]. The surface reactivity of oxide films depends on the number of surface –OH groups. Moreover, the –OH groups on the nanomaterial surface can influence the surface reactivity and wetting [26].

Since hydroxy groups greatly affect the properties of zirconia nanoparticles, detecting their surface concentration and optimizing the synthesis procedures to eliminate or reduce them is of significant technological interest. A number of methods have been developed to measure the hydroxy group site density as well as mechanisms of hydroxylation of nanopowder surfaces [27]. These methods include reaction with Grignard reagents, dehydration by heating, and FTIR spectroscopy. FTIR spectroscopy is a very sensitive and versatile method for the detection of hydroxy groups and it may be easily coupled to the task of chemical group characterization of the nanoparticle surface.

Most nanoparticle characterization methods are focused on the NP size distribution. However, the nanoparticle density measured using helium pycnometry provides valuable information about their phase composition, in the same way as density conveys information about the phase composition of bulk materials. The aim of this paper is to present a method for nanoparticle characterization that permits quality assessment based on density measurements, and also invokes a correction resulting from the surface contribution to the density. The novelty of our findings is a correlation between the density and the specific surface area. This interdependence can be used in nanometrology.

## Results and Discussion

### Phase composition and morphology of ZrO_2_ nanopowders

**Figure 1:**
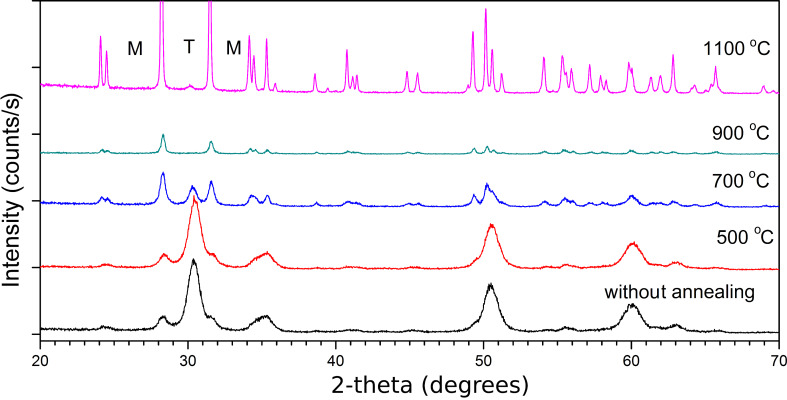
X-ray diffraction patterns of ZrO_2_ synthesized at 5.5 MPa for 20 min and annealed in air for 30 min at the given temperatures. The diffraction peaks assigned to the tetragonal phase and the monoclinic phase are marked as T and M, respectively.

The XRD patterns of ZrO_2_, which was synthesized at 5.5 MPa for 20 min and annealed at 500, 700, 900 and 1100 °C, are presented in Figure 1. It can be seen that powders synthesized with an annealing temperature above 700 °C contained only the monoclinic phase, which was in agreement with known data. Our observations were in agreement with [29–32] where nanocrystalline powders synthesized by hydrothermal techniques contained mainly mixed phases.

**Figure 2:**
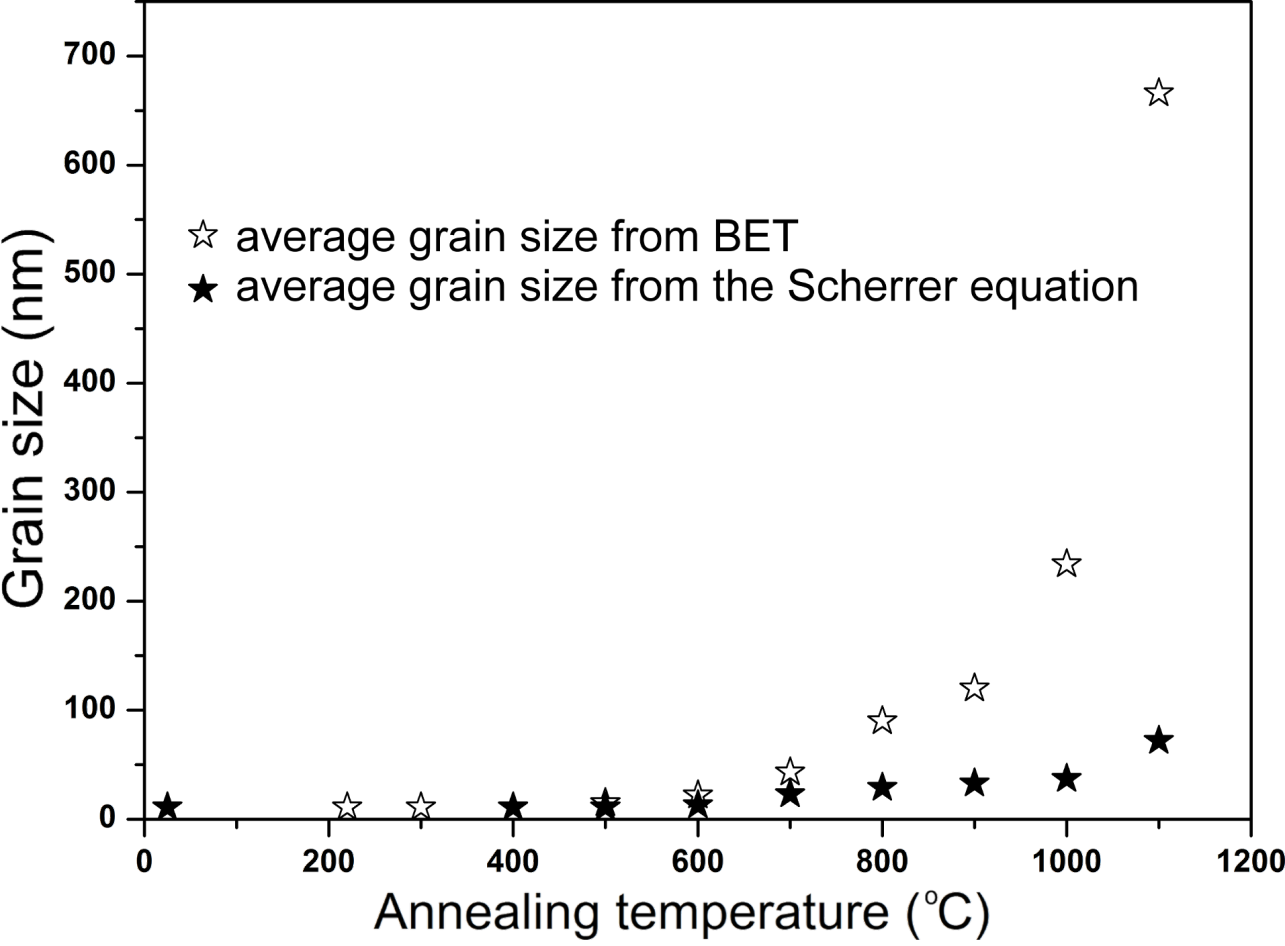
The average grain size of the ZrO_2_ powder as a function of the annealing temperature.

We examined the average grain size of the ZrO_2_ powders as a function of the annealing temperature (Figure 2). The grain size was evaluated by determining the specific surface area obtained from BET analysis and also from the Scherrer equation. Additionally, the average crystallite and grain size, as well as the phase content of the zirconia powders are given in Table 1. Annealing the powders at a temperature lower than 500 °C did not result in an increase in the crystallite size, where the crystallites remained approximately 11 nm in diameter. As the annealing temperature of the nano-ZrO_2_ was increased, the average crystallite and grain size of the powder increased. The presence of agglomerates and particles almost 5 times larger than the nano-ZrO_2_ annealed at 500 °C was found. For powders annealed at 1100 °C, the crystallite diameter increased even further to a diameter of 72 nm (according to the Scherrer equation).

**Table 1:** The average grain size, density and amount of monoclinic phase as a function of the annealing temperature of the nano-ZrO_2_ powders.

Annealing temperature [°C]	Average crystallite size from the Scherrer equation [nm]	Average grain/agglomerate size from BET [nm]	Amount of monoclinic phase [%]	Density [g/cm^3^]^a^

no annealing	11	-	21	-
400	11	11	23	5.22 ± 0.07
500	11	15	17	5.50 ± 0.08
600	13	22	31	5.43 ± 0.09
700	23	43	50	5.58 ± 0.06
800	29	90	100	5.77 ± 0.07
900	33	120	100	5.77 ± 0.05
1000	37	234	100	5.72 ± 0.01
1100	72	666	100	5.87 ± 0.02

^a^SD = 0.05

**Figure 3:**
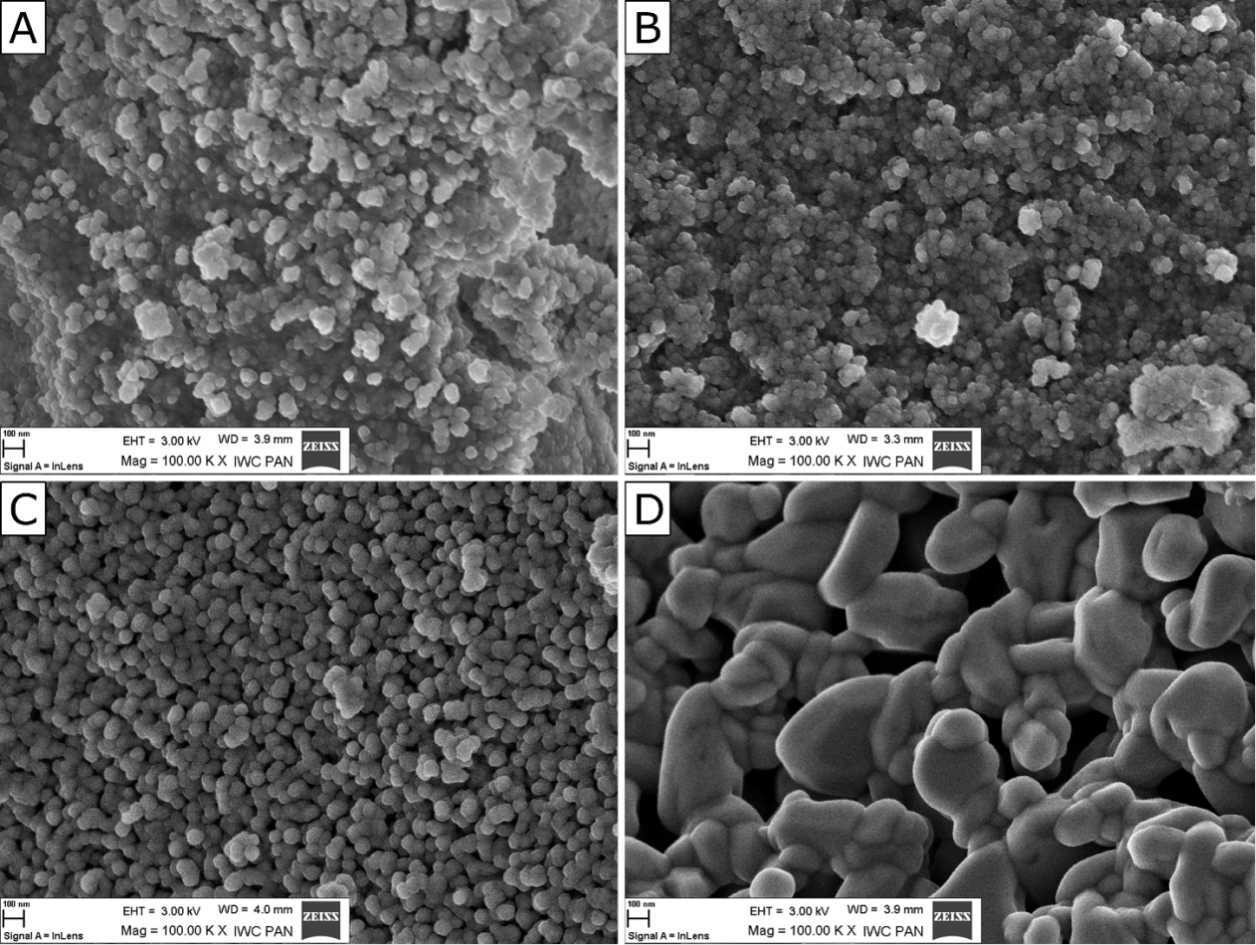
SEM images of four selected nano-ZrO_2_ samples: (A) no annealing, and after annealing at (B) 500 °C, (C) 800 °C and (D) 1100 °C.

The increase in the ZrO_2_ grain size with increasing annealing temperature was also clearly visible in SEM images (Figure 3). The grains were predominately spherical and tended to form agglomerates (Figure 3C). The much higher apparent particle size is the result of particle sintering.

### Characterization of the –OH/–OD groups

**Figure 4:**
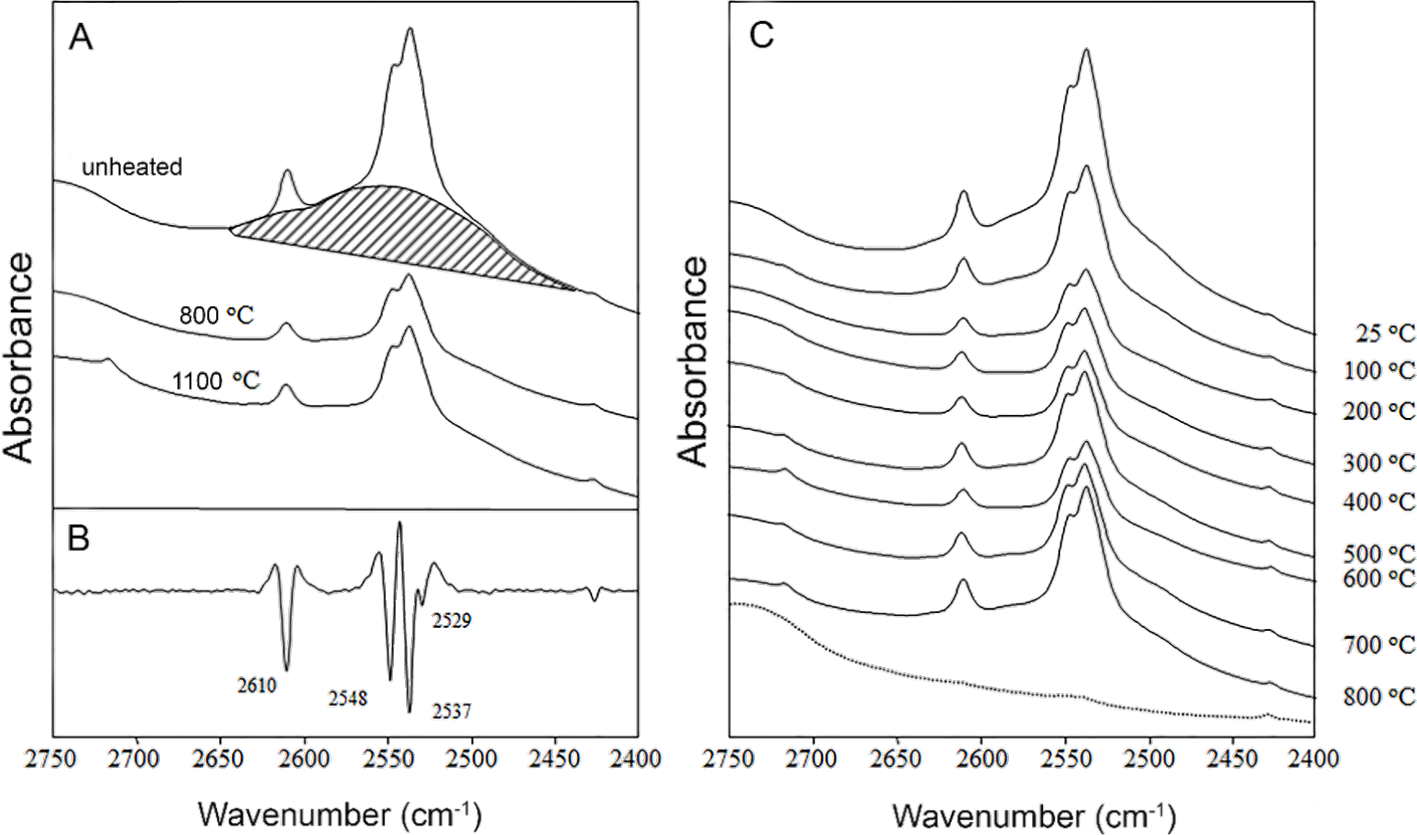
The –OD spectral band in ZrO_2_ samples synthesized in the presence of D_2_O. (A) The stretching vibrations of the –OD groups in the ZrO_2_ samples synthesized in the presence of D_2_O and annealed at 800 °C or 1100 °C. (B) The corresponding second derivative of the spectrum. (C) The temperature dependence of the –OD band. The dotted line represents the spectrum of ZrO_2_ powder obtained in the absence of D_2_O, which therefore lacks –OD stretching vibrations.

The amount of –OH/–OD groups on the nano-ZrO_2_ surface was assessed by FTIR measurements. The –OD spectral bands are present in all ZrO_2_ samples synthesized in the presence of D_2_O (Figure 4). Three major peaks are visible in the original absorption spectra at 2610, 2548, and 2537 cm^−1^ (Figure 4A), although a resolution enhancement by displaying the second derivative reveals another minor band at 2529 cm^−1^ (Figure 4B). It is likely that the four bands correspond to four different populations of –OD groups and they are likely to reflect different surface sites where the –OD groups are attached. The –OD stretching band is in an otherwise “empty” spectral region, as illustrated by the corresponding spectrum of a H_2_O-only preparation of ZrO_2_ (the dotted line in Figure 4C). The well-defined splitting of the –OD band into four separate peaks suggested a highly structured molecular environment of the hydroxide groups, with little hydrogen bonding. This result is opposed to the very broad and featureless –OD band typical for solvents (such as D_2_O) or fast-interchanging hydrogen bonds. The top spectra in Figure 4A and Figure 4C (corresponding to the unheated ZrO_2_ sample) clearly indicate the presence of such a broad, featureless –OD band, which disappeared upon heating to 100–200 °C. This band reflects molecular water adsorbed on the nanoparticle surface or between nanoparticles, and disappears upon drying of the nanopowder. The spectral contribution of these “low-order” –OD groups appears as the area below the three major peaks in Figure 4A. The subsequent spectra, recorded at increasing temperatures (Figure 4C), revealed that the well-ordered, surface –OD groups remained even at 1100 °C (Figure 4C and Figure 4A). A spectral band at 1250 cm^−1^, characteristic of the molecular D–O–D deformation, was absent in the spectra of all ZrO_2_ samples. This indicates that the observed vibrations stem from –OD groups covalently bound to zirconia, rather than from trapped D_2_O molecules.

The stability of –OH groups on the surface of nano-oxides annealed under various temperatures has also been observed by other authors [23,27]. The decomposition of the Zr–OD/OH groups on the NP surface leads to formation of unsaturated Zr bonds. Therefore, contrary to conventional phase diagrams, the alternative to decomposition is not creation of molecules with saturated bonds, but rather, generation of broken bonds. This leads to the thermodynamic stability of the surface layers in contrast to bulk materials, where a phase transformation would occur under the given conditions [33].

**Figure 5:**
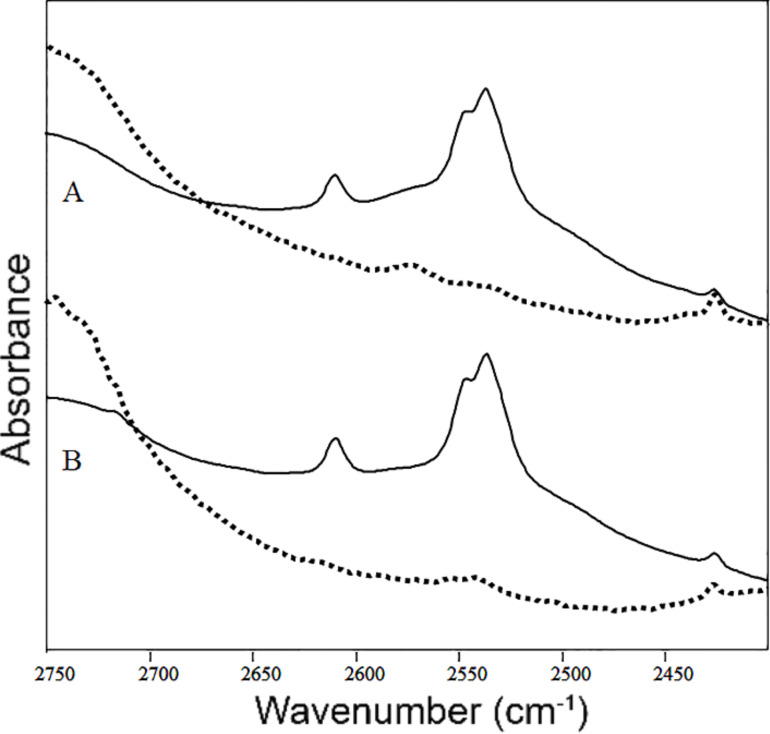
The infrared spectra of the ZrO_2_ samples prepared in the presence of D_2_O and subsequently dried in vacuum at 25 °C (A) or at 800 °C (B). The dotted lines show the spectra after the samples were immersed for 60 min in H_2_O, following the drying process.

ZrO_2_ samples were prepared in the presence of D_2_O and subsequently dried in vacuum at 25 °C or at 800 °C, followed by immersion in H_2_O for 60 min. A comparison of the FTIR spectra of the nano-ZrO_2_ before and after immersion in water (Figure 5) showed a simultaneous increase of the spectral absorption above 2700 cm^−1^ with an even larger –OH band at 3400 cm^−1^ (not shown), which gained intensity at the expense of –OD. These data suggests that the –OD groups are on the surface and were readily exchangeable with –OH groups from water. It was observed that after immersion in water the signal from –OD disappears. This was observed for both the annealed and non-annealed sample. The disappearance of the –OD signal is evidence that the –OD groups were situated on the surface of the grains. Upon contact with water, the –OD groups on the grain surface were replaced by –OH from water. When the samples were dried and measured again, no –OD signal was detected. If the D^+^ ions were situated in interior of the nanoparticle, they would not be replaced by H^+^, and the signal would not change. This experiment provides evidence that the ^−^OD or ^−^OH ions, which are situated on the NP surface, contribute to a reduction of the density of the nanoparticles.

### Nano-ZrO_2_ density in relation to specific surface area

The FTIR results confirmed that the synthesis of zirconia under hydrothermal conditions produced nanopowders coated with a surface layer of –OH groups. Annealing the powders at temperatures up to 500 °C did not cause an increase in the crystallite size. Annealing at a temperature of 200 °C converted the amorphous material to crystalline, which was attributed to the evaporation of water adhered to the nanoparticles surface. The presence of clearly distinguishable peaks above that temperature indicates that the remaining hydroxy groups are for well-defined chemical bonds in the deeper layers of the zirconia NPs. An increase in the annealing temperature caused the crystallite diameter to increase to 72 nm (according to the Scherrer equation). The much larger apparent particle size indicated by the specific surface area measurements is the result of particle sintering. That is, the width of the XRD peaks depends on the size of the crystallites, while the specific surface area depends on the exposed surface of the sintered aggregates. During the sintering process, the –OH groups are most likely removed by evaporation of water, and the thus created dangling bonds are presumably saturated by creating chemical bonds between zirconium and oxygen atoms across the grain boundaries.

**Figure 6:**
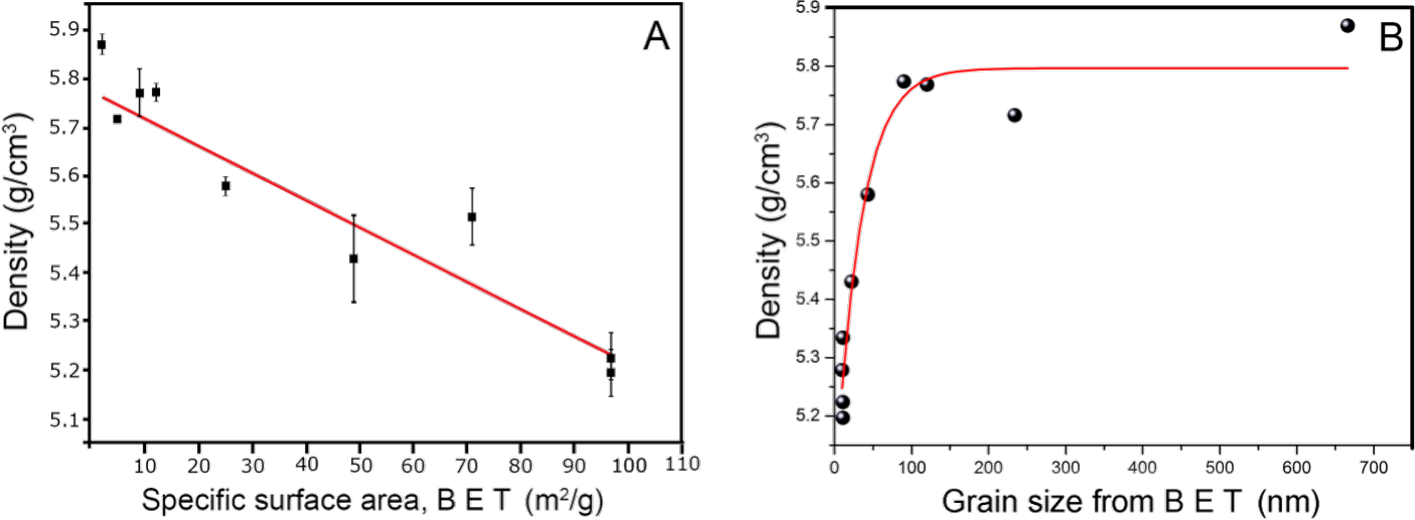
Density as a function of specific surface area (A), experimental and calculated correlation of density as a function of grain size from BET (B) for the nano-ZrO_2_.

The results obtained for the ZrO_2_ nanopowder show a correlation between density and specific surface area (SSA, Figure 6A). It is clear that the greater the average grain size (and thus, the smaller the specific surface area), the greater the density. For very small particles of 11 nm, the SSA is 97 m^2^/g ZrO_2_. At the same time, the density of ZrO_2_ is low at 5.22 g/cm^3^ compared to 5.68 g/cm^3^ for the monoclinic phase of bulk zirconia. Therefore, the growth of the particles causes an increase in the density (Figure 6A), making the density inversely proportional to the specific surface area. This observation is related to the presence of hydroxy groups on the nanomaterial surface. Similar behavior for bulk material was observed by Srdic et al. [34] who investigated the sintering process of nanocrystalline zirconia. The authors found that sintering at 950 °C under vacuum lead to an increase in particle size from 5 nm to 60 nm and an increase of the pellet density, which was attributed to a decrease of total surface of grain boundaries.

The experimental and calculated results of the density as a function of grain size for nano-ZrO_2_ are presented in Figure 6B. Assuming that the surface layer is Zr(OH)_4_, its thickness can be estimated by the fitting function of the effective density versus grain size. The following formula expresses the effective powder density as a function of core/shell densities, their thicknesses, and the grain size:

[1]
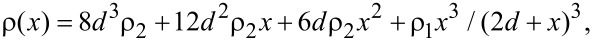


where ρ_1_ is density of the core, ρ_2_ is the density of the shell, x is diameter of the core, *d* is the shell thickness, and ρ is the effective (measured) density of the powder. Given the shell density, one can then calculate the shell thickness, *d*, in nm.

Although the exact composition of the shell is not known, it can be assumed to be composed of hydrogen, oxygen and zirconium atoms. The molecules are bonded with one or two bonds to the nanoparticle surface and the surface layer chemical formula is perhaps Zr(OH)_x_ with x < 4. Since the density of the layer was unknown, a composition of zirconia hydroxide Zr(OH)_4_ was assumed to further the discussion. According to [35], the density of zirconia hydroxide is 3.25 g/cm^3^. Assuming this is the density of the shell, the fitting procedure results in *d* = 0.62 ± 0.07 nm. The diameter of the Zr(OH)_4_ molecule can be estimated to be 0.43 nm. Thus, the thickness of the shell is of the same order of magnitude as the diameter of a zirconium hydroxide molecule. This result is in reasonable agreement with the idea of a monolayer of hydroxide bound to the nanoparticle surface, as observed during FTIR investigations. A surface shell with a slightly lower density than the bulk hydroxide (i.e., 2.80–3.0 g/cm^3^) would result in an exact match of the theoretically calculated and the measured shell thickness. The density of the hydroxide shell layer could possibly be calculated using computer simulation methods.

**Figure 7:**
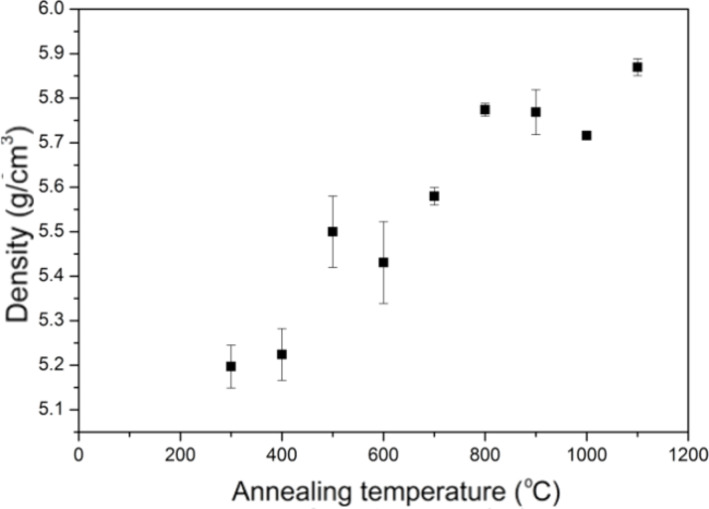
Density as a function of annealing temperature of nano-ZrO_2_ powder.

This work presents a simple model to explain the correlation between the annealing temperature of nano-ZrO_2_ and its density (Figure 7). The density of nano-ZrO_2_ with a grain size of about 10 nm is 10% less than the theoretical value for this oxide.

The density of the powder with grain size 100 nm is close to that of the single crystal. Thus, the variation of density with particle size is a classical nano effect, where new phenomena appear at particle sizes less than 100 nm.

The results presented here are important for nanoparticle metrology and determination of their quality. The smaller the grain size, the larger the contribution of the low density surface layer to the average density. The technological aim is usually to obtain particles with an inconspicuous surface layer, for instance, capped with as few –OH groups as possible. A low-density layer covering the surface of the nanosized particles will lead to a decrease of average density, and this decrease will be more important for small particles as compared to large ones. Ideally, high quality nanoparticles should be situated in the upper right corner of the SSA vs density plot of in Figure 6A, giving the maximum possible density for a given specific surface area.

## Conclusion

It was found that the density of nanoparticles decreases as their size decreases, or as the specific surface area increases. This effect is caused by the surface layer contribution to the average density of the nanoparticles. A surface layer of hydroxy groups is present on the surface of nano-ZrO_2_ particles produced using a hydrothermal process. Many of the hydroxy groups disappear after annealing at 400 °C, however a layer of adsorbed hydroxide remains stable even after heating to 1100 °C and contributes to a reduction in the NP density. It was found that the influence of the surface layer on the NPs density becomes particularly visible for nanoparticles smaller than 50 nm.

The technique of heavy water substitution allowed evaluation of changes in the zirconia surface layers as a function of the processing parameters by using FTIR without the need of in situ measurements in a vacuum chamber. The FTIR spectra of the –OD groups after annealing up to 1100 °C indicate a highly ordered structure of the surface layer with four well-defined and distinct types of –OD molecular bonding. Additionally, the –OD groups located on the surface readily exchange with the –OH groups in the water.

The correlation between density and specific surface area of the nanopowder can be useful for the characterization of nanomaterials and for assessment of their quality. In many cases the technological goal is to avoid amorphous layers and obtain fully crystalline nanoparticles with high density. Nevertheless, because of the surface layers, for a given average NP diameter, a maximum density can be achieved, which is less than for bulk materials.

## Experimental

Nanocrystalline zirconia powders were produced using a microwave-driven hydrothermal process performed at up to 5.5 MPa for 20 min using a previously described procedure [22]. The present synthesis procedure for ZrO_2_ was modified by the substitution of a 50/50 mixture of water (H_2_O) and heavy water (D_2_O) as the synthesis medium. The solution of ZrOCl_2_·8H_2_0 (0.5 M) was adjusted to pH 10 with NaOH (1 M) and poured into a Teflon reaction vessel in the microwave reactor. The microwave reactor (“ERTEC”, Wroclaw, Poland), was operated at 2.45 GHz, and permitted hydrothermal synthesis at pressures up to 10 MPa. After the reaction was complete, the solid phase was filtered from the solution and washed free of dissolved salts with distilled water. The filtered powders were air dried and then annealed in air at temperatures ranging from 25 to 1100 °C for 30 min. Two selected powders, one dried at 25 °C and one annealed at 800 °C, were rinsed for 60 min in H_2_O in order to remove –OD groups, as explained below.

To assess the type and amount of surface layers in the nanoparticles, KBr-based pellets containing 1 wt % ZrO_2_ were prepared for FTIR spectral analysis. FTIR commonly shows a strong infrared absorption around 3400 cm^−1^ that corresponds to stretching vibrations of the –OH bonds. Studies of the annealing effect on –OH groups on the nanoparticle surfaces are typically carried out in vacuum [19,27] since water vapor and –OH groups attached to the surface during synthesis or thermal treatment cannot be distinguished from hydroxy groups formed when the samples are transported in air to the spectrometer. However, the –OD groups observed during our FTIR studies were incorporated into the nanoparticles during synthesis, thus a vacuum system was unnecessary. The –OD stretching vibrations are manifested as a separate band in the infrared spectrum (2600–2400 cm^−1^). This band does not overlap with other infrared peaks and its intensity can be conveniently used as an estimation for the concentration of surface hydroxid groups.

All FTIR spectra were collected in transmission mode using a Nicolet NEXUS FTIR spectrometer equipped with a liquid nitrogen-cooled MCT detector. Each spectrum was the sum of 128 interferograms, with a resolution of 4 cm^−1^. The sample chamber was continuously purged with dry, CO_2_-free air. All data processing, including the calculation of the second derivative Savitzky–Golay spectra, was performed using OMNIC and GRAMS software by Nicolet and ThermoNicolet, respectively.

The determination of the surface area of the powders was carried out using the BET method with a Micromeritics Instruments Gemini 2360 apparatus with nitrogen as the adsorbate. The particle size was calculated based on the BET data, assuming spherical particles, using:

[2]
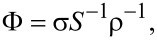


Where σ is the average particle diameter, *S* is the specific surface area of the powder and ρ is the density of zirconia.

The powders were analyzed with an X-ray diffractometer (Cu Kα, Siemens Model D5000). The X-ray diffraction patterns were collected in a 2θ range of 20–90° at room temperature, with a step increment of 0.05°. The Scherrer equation was used to calculate the average crystallite diameter [36].

As previously described [22], the density of the powders was measured using helium pycnometry (Model AccuPyc 1330, Micromeritics Instruments). The in-house procedure for the density measurement was as follows: the powders were flushed in a helium atmosphere for 120 min at 220 °C, weighed, and then transferred to the helium pycnometer. The nanoparticle density can be influenced by air absorbed onto the surface of the nanopowder during transfer to the pycnometer. Therefore, the measurement was repeated up to 100 times, and the density as a function of the number of cycles was plotted. Each cycle partially removed the absorbed water and the density increased. The process was stopped when the results reached an asymptotic value, and this value was taken as the powder density.

The morphology of the powders was examined using a LEO 1530 Scanning Electron Microscope.
